# Supplementary material for: Adipocytes control food intake and weight regain via Vacuolar-type H+ ATPase
Source: Nat Commun. 2022 Aug 30;13:5092. doi: 10.1038/s41467-022-32764-5 (PMC9427743; doi:10.1038/s41467-022-32764-5)

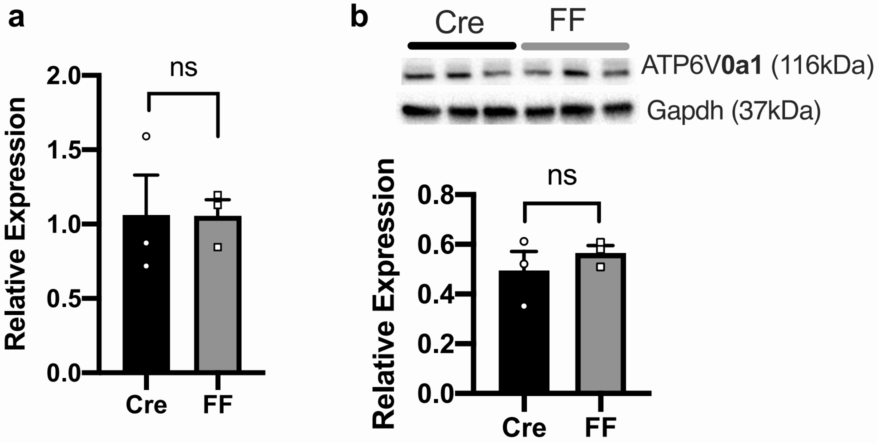

Supplemental figure 1. Atp6V0a1 levels in Cre and FF control mice adipose tissue. A. RNA and (n=3/group; t=0.18, df=4, p=0.49). **B.** Protein levels of Atp6v0a1 in Cre (black bars) and FF (grey bars) controls, (n=3/group; t=0.87, df=4, p=0.22). Data is expressed as mean ± SEM and was analyzed using students t-test (2-tailed).

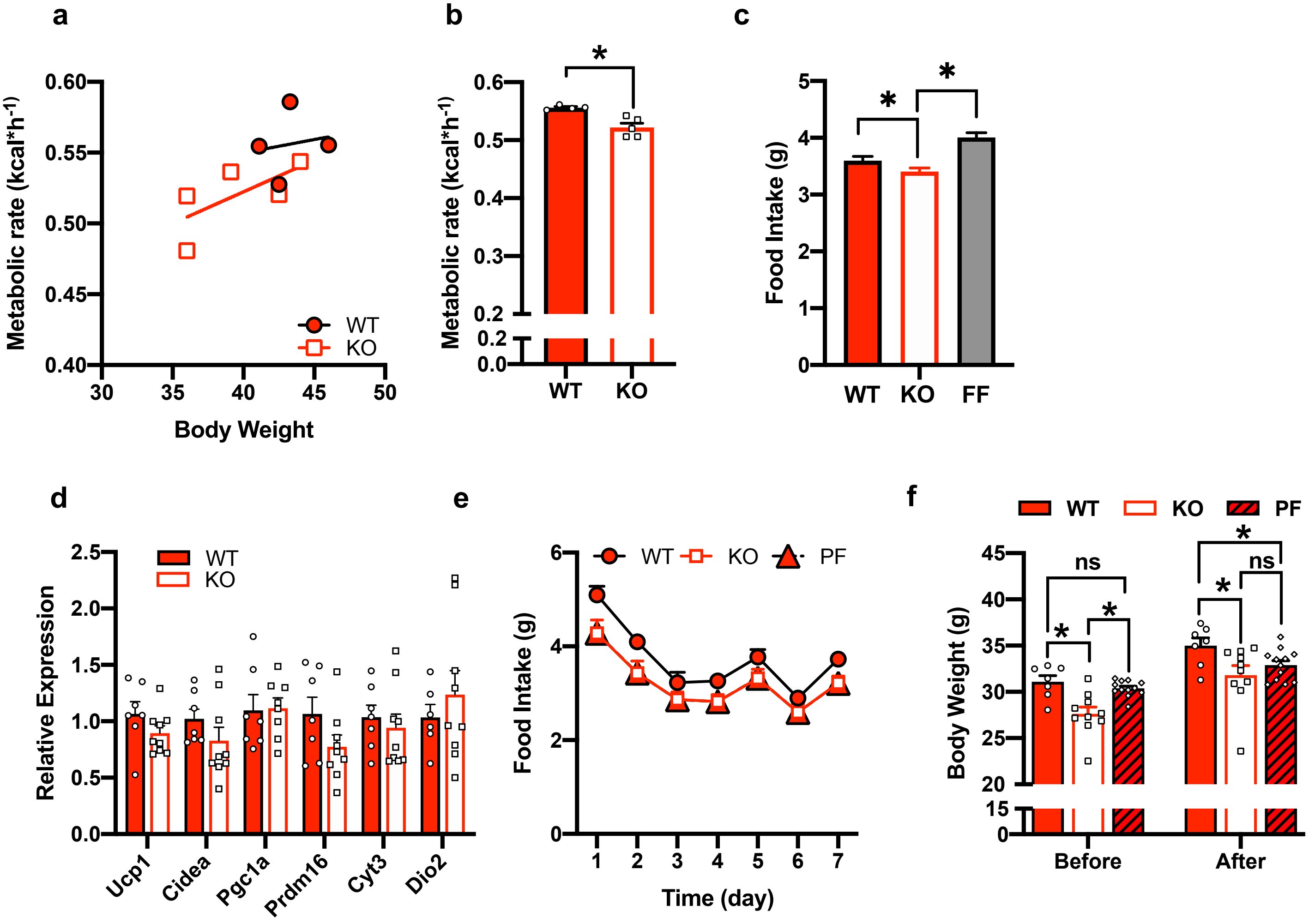

Supplemental figure 2. Adipocyte specific Atp6v0a1 KO mice fed HFD. a. Metabolic rate, WT (red circles, n= 4, KO (white squares, n=5). b. Average metabolic rate, WT (red circles, n= 4, KO (white squares, n=5), p< 0.0001. c. Food intake WT (red bars, n= 7), KO (white bars, n=10) and FF (grey bars, n= 12), p< 0.001. d. BAT relative gene expression (Ucp1: t=1.44, df=14, p=0.17; Cidea: t=1.24, df=14, p=0.23; Pgc1a: t=0.12, df=13, p=0.90; Prdm16: t=1.66, df=14, p=0.12; Cyt3: t=56, df=14, p=0.59; Dio2: t=0.73, df=13, p=0.48), WT (red bars, n= 7), KO (white bars, n=9). e. Food intake in KO (white squares, n=8) and WT ad libitum fed (red circles, n= 7) , and WT pair fed (PF) mice (red triangle, n= 12) (genotype: F=3.44, df=2, p=0.06; time: F=20.28, df=6, p<0.001, genotype X time: F=0.48, df=12, p=0.92), f. Body weight in KO (white bars, n= 8) and WT ad libitum fed (red bars, n=7) , and WT pair fed (PF) mice (red hatched bars, n=12) (group: F=6.06, df=2, p=0.007; time: F=156, df=1, p<0.001, group X time: F=3.944, df=2, p=0.03). Suppl. Fig. 2 a-c were analyzed using ANCOVA with body weight as covariate (genotype: F=4.007, df=2, p<0.001; BW: F=8.109, df=1, p=0.006). Suppl. Fig. 2d was analyzed by student t-test (2-tailed). Suppl. Fig 2e. was analyzed by repeated-measures two-way ANOVA followed by two-stage linear step-up procedure of Benjamini, Krieger and Yekutieli with false discovery rate of 0.10. Suppl. Fig. 2f. was analyzed by one-way ANOVA followed by two-stage linear step-up procedure of Benjamini, Krieger and Yekutieli with false discovery rate of 0.10. * denotes statistical significance at *p* < 0.05.

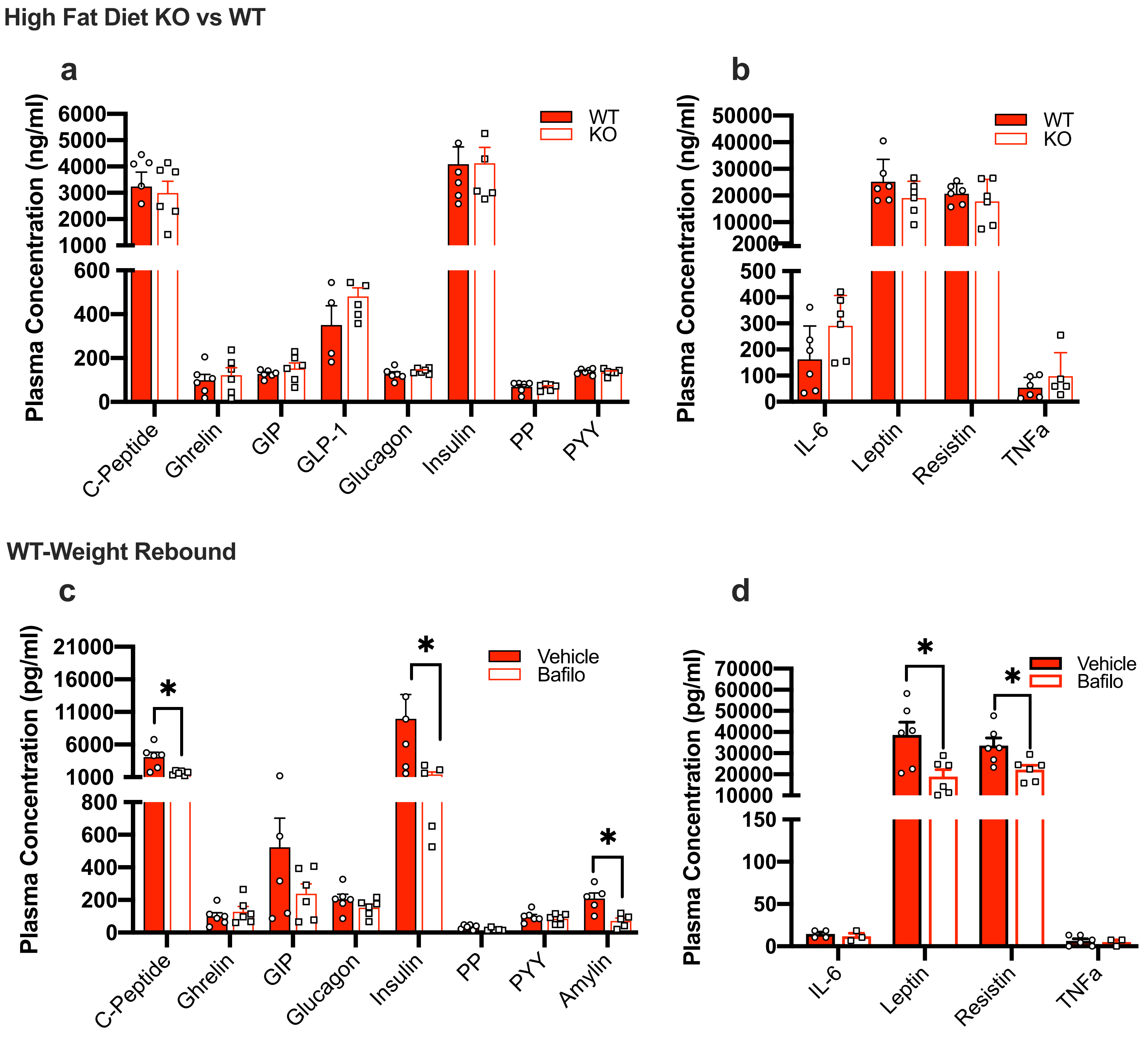

Supplemental Figure 3. Plasma levels of appetite regulating hormones and inflammatory mediators in mice. a. Plasma levels of appetite regulating hormones (C-peptide: t=.34, df=10, p=0.37; Ghrelin: t=0.53, df=10, p=0.30; GIP: t=0.98, df=10, p=0.18; GLP-1: t=1.53, df=8, p=0.08; Glucagon: t=1.26, df=10, p=0.11; Insulin: t=0.04, df=10, p=0.46, PP: t=0,09, df=10, p=0.46; PYY: t=0.33, df=10, p=0.37). WT (red bars, n=6) and KO (white bars, n=6), and b. inflammatory mediators (IL-6: t=1.82, df=10, p=0.04; Leptin: t=1.442, df=10, p=0.09; Resistin: t=0.77, df=10, p=0.23; Tnfa: t=1.08, df=9, p=0.15) in HFD- fed WT (red bars, n=6) and KO (white bars, n=6) mice. c. Plasma levels of appetite regulating hormones (C-peptide: t=3.29, df=10, p=0.004; Ghrelin: t=0.75, df=10, p=0.23; GIP: t=1.52, df=10, p=0.08; Glucagon: t=1.33, df=10, p=0.11; Insulin: t=2.3, df=10, p=0.02, PP: 2.14, df=9, p=0.003; PYY: t=0.73, df=10, p=0.24; Amylin: t=0.3.45, df=8, p=0.004) , vehicle (red bars, n=6) and Bafilo (white bars, n=6), and d. inflammatory mediators (IL-6: t=0.67, df=5, p=0.26; Leptin: t=2.86, df=10, p=0.005; Resistin: t=2.75, df=10, p=0.01; Tnfa: t=0.34, df=7, p=0.37) in WT mice treated with vehicle (red bars, n=6) or bafilomycin (white bars, n=6). Data is expressed as mean ± SEM and was analyzed by student t-test (2-tailed). * denotes statistical significance at p = 0.05.

Supplemental Figure 1 – full blots

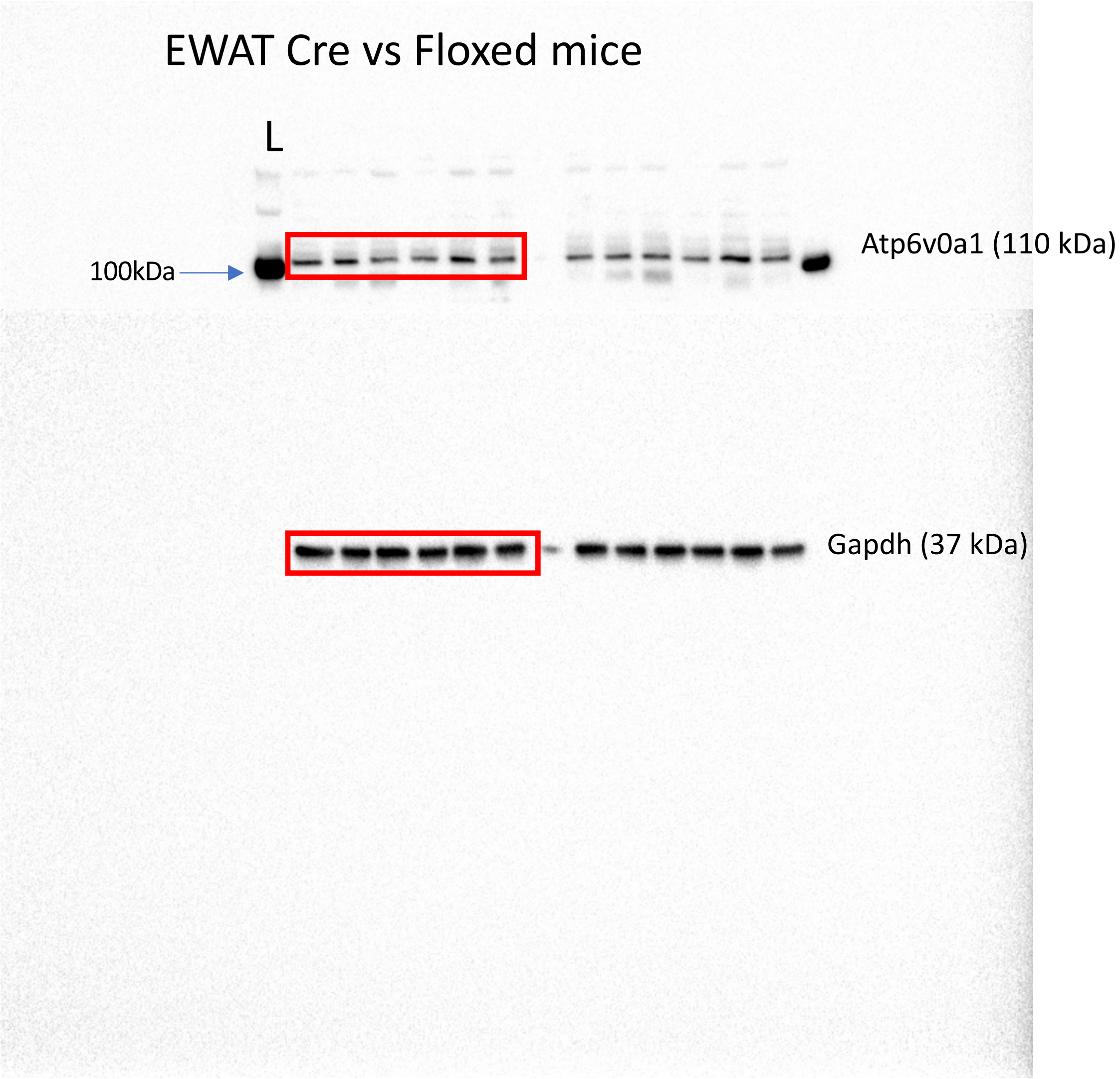

Supplement: Supplementary file 1 — Supplementary Information [file 41467_2022_32764_MOESM1_ESM.pdf]
