# Supplementary material for: Adipocytes control food intake and weight regain via Vacuolar-type H+ ATPase
Source: Nat Commun. 2022 Aug 30;13:5092. doi: 10.1038/s41467-022-32764-5 (PMC9427743; doi:10.1038/s41467-022-32764-5)
Supplement: Supplementary file 3 — Description of Additional Supplementary Files [file 41467_2022_32764_MOESM3_ESM.pdf]

Description of Additional Supplementary Files

File Name: Supplementary Data 1
Description: Obesity-induced adipocyte genes that were persistently dysregulated genes after weight loss.

File Name: Supplementary Data 2
Description: Obesity-induced SVF genes that were persistently dysregulated genes after weight loss.

File Name: Supplementary Data 3
Description: C. elegans orthologs of mouse metabolic memory genes.

File Name: Supplementary Data 4
Description: Food intake in C. elegans mutants compared with control ‘wild type’ N2 strain.

File Name: Supplementary Data 5
Description: C. elegans mutant food intake response to serotonin treatment.

File Name: Supplementary Data 6
Description: Human orthologs of mouse metabolic memory genes that were also significantly differentially expressed in obese human adipocytes. Human adipocyte data obtained from GEO/ GSE2508, https://www.ncbi.nlm.nih.gov/geo/query/acc.cgi?acc=GSE2508, and analyzed with GEO2R[78] using default parameters including P values adjusted by Benjamini & Hochberg (False Discovery Rate)

File Name: Supplementary Data 7
Description: Primer sequences used in the qPCR.
