# Supplementary material for: Adipocytes control food intake and weight regain via Vacuolar-type H+ ATPase
Source: Nat Commun. 2022 Aug 30;13:5092. doi: 10.1038/s41467-022-32764-5 (PMC9427743; doi:10.1038/s41467-022-32764-5)
Supplement: Supplementary file 5 — Reporting Summary [file 41467_2022_32764_MOESM5_ESM.pdf]

nature portfolio

Corresponding author(s): Osborn , O

Last updated by author(s): 8/10/2022

Reporting Summary

Nature Portfolio wishes to improve the reproducibility of the work that we publish. This form provides structure for consistency and transparency in reporting. For further information on Nature Portfolio policies, see our Editorial Policies and the Editorial Policy Checklist.

Statistics

For all statistical analyses, confirm that the following items are present in the figure legend, table legend, main text, or Methods section.

n/a	Confirmed	
☐	☒	The exact sample size (*n*) for each experimental group/condition, given as a discrete number and unit of measurement
☐	☒	A statement on whether measurements were taken from distinct samples or whether the same sample was measured repeatedly
☐	☒	The statistical test(s) used AND whether they are one- or two-sided<br>*Only common tests should be described solely by name; describe more complex techniques in the Methods section.*
☐	☒	A description of all covariates tested
☐	☒	A description of any assumptions or corrections, such as tests of normality and adjustment for multiple comparisons
☐	☒	A full description of the statistical parameters including central tendency (e.g. means) or other basic estimates (e.g. regression coefficient) AND variation (e.g. standard deviation) or associated estimates of uncertainty (e.g. confidence intervals)
☐	☒	For null hypothesis testing, the test statistic (e.g. *F*, *t*, *r*) with confidence intervals, effect sizes, degrees of freedom and *P* value noted<br>*Give P values as exact values whenever suitable.*
☒	☐	For Bayesian analysis, information on the choice of priors and Markov chain Monte Carlo settings
☒	☐	For hierarchical and complex designs, identification of the appropriate level for tests and full reporting of outcomes
☒	☐	Estimates of effect sizes (e.g. Cohen's *d*, Pearson's *r*), indicating how they were calculated

Our web collection on statistics for biologists contains articles on many of the points above.

Software and code

Policy information about availability of computer code

Data collection	No software was used for data collection
Data analysis	Sequence reads were first mapped to the mouse transcriptome using Bowtie2 algorithm and counted as reads per gene using RSEM and then analyzed using the statistical algorithm limma. Statistics were carried out using GraphPad 8.1. Metabolic chamber data was processed using CLAMS data eXamination (CLAX) software (Columbus Instruments).

For manuscripts utilizing custom algorithms or software that are central to the research but not yet described in published literature, software must be made available to editors and reviewers. We strongly encourage code deposition in a community repository (e.g. GitHub). See the Nature Portfolio guidelines for submitting code & software for further information.

Data

Policy information about availability of data

All manuscripts must include a data availability statement. This statement should provide the following information, where applicable:

- Accession codes, unique identifiers, or web links for publicly available datasets
- A description of any restrictions on data availability
- For clinical datasets or third party data, please ensure that the statement adheres to our policy

All data are available in the main text or the supplementary materials. The RNA seq data generated in this study have been deposited in the GEO database under accession code GSE210014. The processed RNA data generated in this study are provided in the Supplementary tables 1 and 2.

Human research participants

Policy information about studies involving human research participants and Sex and Gender in Research.

Reporting on sex and gender	Sex was determined on self-reporting. Sex was not considered in the design of the study related to humans. The non-obese group contained 2 females and 2 males, the participants with obesity group contained 2 females and 3 males. Sex-based analysis was not performed due to the limited sample size.
Population characteristics	Subjects providing samples for analysis in the current study were recruited under several IRB-approved projects. The samples for the current study were selected from a bio-repository on the basis of body mass index (<27.0 kg/m2, or >32), blinded to sex, age or clinical diagnosis. Subjects analyzed in the current study ranged in age from 37-64 yrs and included 4 females and 5 males.
Recruitment	Participants were recruited from the community (newspaper advertisements, and from clinics and physicians within the VA/ UCSD healthcare systems. The recruitment population reflected the demographics of the San Diego area.
Ethics oversight	The projects were approved by the Institutional Review Boards of the Veterans Affairs San Diego Healthcare System and the University of California, San Diego.

Note that full information on the approval of the study protocol must also be provided in the manuscript.

Field-specific reporting

Please select the one below that is the best fit for your research. If you are not sure, read the appropriate sections before making your selection.

☒ Life sciences ☐ Behavioural & social sciences ☐ Ecological, evolutionary & environmental sciences

For a reference copy of the document with all sections, see nature.com/documents/nr-reporting-summary-flat.pdf

Life sciences study design

All studies must disclose on these points even when the disclosure is negative.

Sample size	Sample sizes were selected based on previous similar studies, PMID: 30532051, PMID: 34294678.
Data exclusions	No data were excluded in this study
Replication	Food intake and body weight studies have been replicated in multiple cohorts. All attempts at replication were successful.
Randomization	Animal Body weights were matched across drug/vehicle groups at baseline to facilitate measurement of drug induced changes in body weight and metabolism across groups (Fig 6 &7)
Blinding	In this preclinical animal study blinding was not conducted as the majority of in vivo studies were conducted by one postdoctoral fellow

Reporting for specific materials, systems and methods

We require information from authors about some types of materials, experimental systems and methods used in many studies. Here, indicate whether each material, system or method listed is relevant to your study. If you are not sure if a list item applies to your research, read the appropriate section before selecting a response.

Materials & experimental systems

n/a	Involved in the study	
☐	☒	Antibodies
☐	☒	Eukaryotic cell lines
☒	☐	Palaeontology and archaeology
☐	☒	Animals and other organisms
☐	☒	Clinical data
☒	☐	Dual use research of concern

Methods

n/a	Involved in the study	
☒	☐	ChIP-seq
☒	☐	Flow cytometry
☒	☐	MRI-based neuroimaging

Antibodies

Antibodies used	pan-Akt (Cell signaling #4691) Cell signaling website shows specificity using a blocking peptide. Cited 3616 times in the literature.<br>pSer473-AKT (Cell signaling 406), Cell signaling website shows specificity using lamda phosphatase inhibitors, PI3K inhibitor (LY294002) as well as insulin treatment.<br>Beta Actin (Cell Signaling 3700). Cited 2859 times in the literature.<br>ATP6v1a (Abcam #199325), cited twice, PMID: 31068699, PMID: 30962575.<br>ATP6v0a1 (Antibody Verify (#AAS81465C), validated in KO, (This paper)<br>Caveolin (BD Transduction Lab #610407), 56 citations. BD website states 'This antibody is routinely tested by Western blot analysis and immunofluorescent imaging.<br>Gapdh (Cell Signaling #5174), siRNA knockdown shows specificity PMID: 27489353<br>Anti-mouse IgG (Jackson ImmunoResearch #115035003),<br>Anti-rabbit IgG (GE Healthcare #NA934V).
Validation	Validation of ATP6v0a1 antibody in KO/WT, figure 2.

Eukaryotic cell lines

Policy information about cell lines and Sex and Gender in Research

Cell line source(s)	3T3L1 adipocytes, ATCC
Authentication	authenticated by ATCC. Tested for mycoplasma contamination using Hoechst DNA stain, Agar culture method and PCR-based assay.
Mycoplasma contamination	We routinely checked all our cell lines for mycoplasma using a PCR assay.
Commonly misidentified lines<br>(See ICLAC register)	N/A

Animals and other research organisms

Policy information about studies involving animals; ARRIVE guidelines recommended for reporting animal research, and Sex and Gender in Research

Laboratory animals	Wild type, Adiponectin cre and ATP6v0a1 Floxed mice , and adipocyte specific ATP6v0a1 KO mice were used in this study. All mice were on a C57BL6 background. All mice were 9-10 weeks of age at the start of the studies.
Wild animals	None
Reporting on sex	Male mice were used in this study as they have further impaired glucose tolerance and insulin resistance compared with matched female mice.
Field-collected samples	None
Ethics oversight	All experiments were approved by and conducted in accordance with the University of California, San Diego IACUC, protocol number S18121.

Note that full information on the approval of the study protocol must also be provided in the manuscript.

Clinical data

Policy information about clinical studies

All manuscripts should comply with the ICMJE guidelines for publication of clinical research and a completed CONSORT checklist must be included with all submissions.

Clinical trial registration	Samples from human subjects analyzed in this study were collected under multiple IRB-approved protocols. These protocols were iniated either before registration in ClinicalTrials.gov was required or did not meet the definition of a clinical trial.
Study protocol	Full protocols for the studies involved are stored on the restricted access Research drive of the VA San Diego Healthcare System. As mentioned above, none of the protocols involved were registered on ClinicalTrials.gov.
Data collection	Samples and data were collected at the VA San Diego Special Diagnostics and Treatment Unit and the UCSD General Clinical Research Center. First subject was enrolled on 03/2001. Last clinical data and sample was collected on 05/2005.
Outcomes	Samples were collected for in vitro analysis; protein expression, glucose transport activity. Metabolic phenotyping was performed either by an oral glucose tolerance test or a hyperinsulinemic/euglycemic clamp procedure.
